# Sedentary Habits

**Published:** 1876-03

**Authors:** 


*Sedentary habits*, or the sedentary occupations of life, greatly tend to constipation and inactivity of the bowels — hence men of literary pursuits, or those closely occupied in study, as students, or employed at the desk or counter, as clerks and accountants; or those who are confined to seats, as tailors, seamstresses, milliners, etc., are all extremely liable to suffer greatly from confined bowels. The want of bodily exercise generally lessens the demand for food, weakens the digestive organs, and indigestion and constipation are almost a necessary consequence. Witness the large number of poor young women in New-York who sit and ply the needle from daylight in the morning till ten or eleven o'clock at night, with scarcely intermission enough to take their meals, or to attend to the calls of nature. This employment, so unremitting in exercise, continues from Monday morning to Saturday night *in one wearying, unvarying sedentary position.* No class of persons in this metropolis are so deserving of sympathy and commiseration as these poor women. Spirit of Tom Hood, look down upon these *white slaves* and pity them. Well didst thou sing:

"'O men with children dear!
O men with sisters and wives!
It is not *linen* you're wearing out,
But human creatures' lives!
Stitch, stitch, stitch,
In poverty, hunger and dirt,
And still she sews with a double thread
A shroud as well as a shirt.'

How many sing that '*song of the shirt*' in New-York to-day whilst they sew their shrouds?

Some of the causes of constipation among the richer and higher classes of people are the modern and irregular hours of society, including late breakfasts, late dinners, and all the long nocturnal pastimes of music and dancing in crowded and heated rooms, etc.; all of which necessarily imply many contraventions and restraints of the laws and of the regular discharge of the functions of nature.

The best remedy is "Nelaton's Suppository" for Piles and Constipation.

